# Exploring Maladaptive Eating Behaviors and Quality of Life in Those with Bowel Diseases

**DOI:** 10.3390/nu17233738

**Published:** 2025-11-28

**Authors:** Lauren Kness, Virginia Quick

**Affiliations:** Department of Nutritional Sciences, School of Environmental & Biological Sciences, Rutgers University, New Brunswick, NJ 08901, USA

**Keywords:** maladaptive eating, disordered eating, young adults, irritable bowel syndrome, inflammatory bowel disease, quality of life

## Abstract

**Background:** Young adults with bowel conditions—such as irritable bowel syndrome and inflammatory bowel diseases—often experience poor quality of life, which may be influenced by their disease management, including eating behaviors. This study aimed to explore maladaptive eating behaviors and quality of life among young adults diagnosed with bowel conditions (IBS, Crohn’s, and Ulcerative Colitis), stratified by gender. **Methods:** A cross-sectional online survey assessing Quality of Life (QOL) and maladaptive eating behaviors (EDE-Q, TFEQ-18) was conducted in 73 young adults with bowel conditions (70% women; mean age 25.16 ± 2.94 years) in 2022/2023. **Results:** Participants reported an average of 10.72 ± 7.46 SD physically or mentally unhealthy days in the past month, indicating poor perceived health status. Mean EDE-Q global scores were higher than published normative data for healthy young adults (men: 2.49 ± 1.26 SD vs. 0.95 ± 0.98 SD; women: 2.30 ± 1.12 SD vs. 1.65 ± 1.30 SD). Mann–Whitney U tests revealed no significant gender differences in QOL or EDE-Q scores. However, men (*n* = 22) reported self-induced vomiting (68.18% vs. 29.41%) and medicine misuse (63.64% vs. 37.25%) ≥ 4 times in the past month at a higher proportion than women (*n* = 51). After controlling for age at diagnosis, the Global EDE-Q score was positively associated with QOL Global score (*r_partial_* = 0.534, *p* < 0.001) and with the number of unhealthy days reported in the past month (*r_partial_* = 0.452, *p* < 0.001) indicating a relationship of moderate strength. **Conclusions:** These findings highlight the potential value of incorporating routine screening for eating disorder risk into the medical treatment of young adults with bowel conditions.

## 1. Introduction

Bowel conditions, such as inflammatory bowel diseases (i.e., Crohn’s disease and ulcerative colitis) and irritable bowel syndrome (IBS), are frequently diagnosed in youth and young adults [1]. Managing and coping with bowel conditions at any life stage presents physical and mental challenges, but these challenges can be particularly painful and disabling for young people [2].

Globally, IBS affects between 10 and 25 percent of the population [1,3]. Women are 1.5 to 3 times more likely to have IBS than men [1,4]. IBS is a functional disorder of the gastrointestinal (GI) system with unclear pathophysiology. Patients typically experience abdominal pain and altered bowel habits as well as abdominal bloating/distension [1,3]. Daily stress and psychological distress appear to worsen GI symptoms, so relaxation techniques and stress management offer promising improvements in symptoms [3]. Diet modification has also been considered as a treatment option with some success using exclusion diets [5].

An estimated 3.1 million people are diagnosed with an inflammatory bowel disease (IBD) in the U.S. [6], and the number of cases appears to be growing [7,8]. IBD includes Crohn’s disease and ulcerative colitis, which are autoimmune diseases characterized by chronic inflammation of the GI tract with unknown etiology [8]. Crohn’s disease can cause inflammation throughout the entire digestive tract, whereas ulcerative colitis causes inflammation localized to the colon [7]. IBD etiology is thought to be multifactorial, stemming from environmental, genetic, and microbiological influences [9]. Currently, no cure exists for IBD, but treatment options can help reduce symptoms and place IBD into remission [7]. Anti-inflammatory drugs like corticosteroids are commonly used to treat symptoms, as well as dietary modification when flare ups occur [7]. However, there are potential negative side effects from these treatments, such as vomiting, diarrhea, and nausea, that can be debilitating. Additionally, changes to physical appearance such as acne and cushingoid features are other potential negative side effects from treatment that can lead to negative body image and self-esteem [7].

Some foods are associated with reoccurring GI symptoms in patients with IBS and IBD, which makes mealtimes stressful [8,10]. Those with bowel conditions may be prone to developing maladaptive eating behaviors and negative body image due to the pressures of managing their disease [11]. Maladaptive eating behaviors include a preoccupation with food and body shape, concern over mealtimes, food restrictions, binge eating, and compensatory behaviors such as self-induced vomiting. While there is limited evidence to suggest that people with IBD or IBS are more likely to have eating disorders, negative eating-related practices are common in this population [12]. For instance, a systematic scoping review reported a high occurrence of self-reported food avoidance and restrictive dietary behaviors (e.g., fasting, skipping meals, rigid food rituals) among IBD patients [13]. Among 955 IBS patients in a cross-sectional study, 13.2% reported severe food avoidance and restriction as well as low quality of life and severe psychological and GI symptoms [14]. Additionally, the increased pressure women face in society to maintain a certain body weight and shape may place them at greater risk for maladaptive eating behaviors compared to men [15]. Prior literature suggests women with IBS have less body appreciation and greater self-criticism compared to healthy controls, which may lead to further elevations in psychological distress [16]. The unpredictability of bowel-related symptoms can also place additional pressure on women to control their bodies, increasing their eating disorder risk. Thus, GI symptoms present in those with bowel conditions may result in feelings of bodily shame, particularly among women, leading to low self-esteem and maladaptive eating behaviors that may influence QOL [12].

Quality of Life (QOL) is a multifactorial construct and has been defined by the World Health Organization as “an individual’s perception of their position in life in the context of the culture and value systems in which they live and in relation to their goals, expectations, standards and concerns.” [17]. Previous literature has found that children and adults with bowel conditions such as IBS and IBD have significantly lower QOL compared to their healthy counterparts [18,19]. Additionally, anxiety and depression severity are heightened in those with bowel conditions and can interact bidirectionally with symptoms, ultimately exacerbating disease severity and negatively affecting QOL [20,21,22]. QOL score differences by gender among IBD adults are mixed in the literature [20]. Some evidence suggests that women have poorer QOL than men [23,24,25], while other studies have found no QOL differences by gender [26,27,28]. Gender differences in adults with IBS suggest that women are particularly at risk for more fatigue, depression, anxiety, and less positive well-being and self-control than men [4]. Women with IBS are also more likely to struggle with negative body image, health worries, and sexual health problems compared to men with IBS [4].

Given the negative impact that bowel conditions may have on eating behaviors, body image, and QOL among young adults, comprehensively examining maladaptive eating behaviors and QOL in this population is an important component of healthcare treatment [29]. A bowel condition may affect an individual’s daily activities, academic performance, ability to work, and social life. Thus, exploring QOL domains such as body image and food avoidance alongside maladaptive eating behaviors in a comprehensive manner may help to improve the care provided to individuals with IBS and IBD [29]. The present study aims to explore QOL and maladaptive eating behaviors among young adults with IBS and IBD by gender.

## 2. Materials and Methods

This is a secondary analysis of a cross-sectional study (2022/2023) that was repeated from an original study conducted 13 years earlier [30,31]. Participants were recruited for this repeated cross-sectional study from February 2022 to December 2023 for a cross-sectional trend analysis. However, for the purposes of this secondary analysis, only data collected from 2022/2023 were included. This study was performed in line with the principles of the Declaration of Helsinki. The Institutional Review Board (IRB) at Rutgers University granted approval of this study. All participants gave informed consent before completing the online survey.

### 2.1. Recruitment

A convenience sample of young adults aged 18–30 years were recruited to take an online survey of eating behaviors and attitudes using email listservs, announcements in courses, and online/social media posts (i.e., Facebook, Instagram, Reddit) targeting young adults with and without diet-related chronic diseases. The inclusion criteria were being 18–30 years of age and having access to the Internet to complete the online survey. However, for the purposes of this secondary analysis, only young adults with IBD (Crohn’s disease and ulcerative colitis) and IBS were included in this exploratory study. As an incentive, participants were entered to win 1 of 5 online $20 gift cards if they provided their name and email address at the end of the online survey, which was kept confidential. The online gift card winners were chosen through a random number generator and emailed at the email address provided in the survey.

The online survey was available for participants to complete using a personal device (personal computer, tablet, smartphone, etc.) that had access to Qualtrics^®^ (Provo, UT, USA) over the Internet, Qualtrics^®^ being the software platform used for the survey. The online survey took approximately 45–60 min for participants to finish; however, there was no time limit, so participants were able to use as much time as they needed to complete the survey.

### 2.2. Measures

This cross-sectional study is a replication of an original study that can be found in greater detail elsewhere [30,31]. The original study examined psychographic characteristics and disordered eating behaviors among young adults with and without diet-related chronic health conditions. Selected measures used in this repeated cross-sectional study in 2022/2023 that were specific to bowel diseases are discussed below.

#### 2.2.1. Sociodemographic and Health Characteristics

Sociodemographic characteristics such as gender, age, race, and year in college were examined along with health-related characteristics such as self-reported heights and weights to calculate body mass index.

#### 2.2.2. Quality of Life Characteristics

Issues related to Quality of Life (QOL) for specific diseases can be assessed through disease-specific QOL instruments. For this study, a modified version of several reliable and validated QOL measurements was created to develop a disease-specific QOL instrument with 35 questions on a 5-point Likert scale [30]. The disease-specific QOL instrument was modified from the diabetes QOL brief clinical inventory [32], celiac disease questionnaire [33], Cystic Fibrosis QOL [34], short inflammatory bowel disease questionnaire [35], and IBS-QOL measures [36]. The developed instrument assesses one’s overall QOL within the past few weeks and has fairly good internal reliability [30]. The items were modified slightly to make it applicable to each diet-related chronic disease. Two nutrition experts also reviewed the QOL items for clarity and content validity during instrument development. The disease-specific QOL instrument has six subscales that are described further below.

The Dysphoria/Emotional scale has 10 items on a 5-point scale (1 = never, 2 = seldom, 3 = sometimes, 4 = often, and 5 = all the time) that evaluate how often an individual feels bad about their health condition (e.g., During the past few weeks, how often has your [name of bowel condition] made you feel depressed?). Items were averaged, with higher mean scores indicating greater emotional vulnerability.

The Interference with Activity of Physical Functioning scale, with 8 items on a 5-point scale (1 = never, 2 = seldom, 3 = sometimes, 4 = often, and 5 = all the time), assesses an individual’s physical ability and mobility (e.g., During the past few weeks, my [name of bowel condition] affected the time I was able to spend doing light tasks like preparing a snack or walking around.) Items were averaged, with higher mean scores indicating greater interference with activities.

The Food Avoidance scale has 3 items on a 5-point scale (1 = strongly disagree, 2 = disagree, 3 = neither, 4 = agree, 5 = strongly agree) that assess the avoidance of food (e.g., During the past few weeks, my [name of bowel condition] has made me feel frustrated because I could not eat when I wanted.). Items were averaged, with higher mean scores indicating greater food avoidance.

The Body Image scale has 2 items on a 5-point scale (1 = strongly disagree, 2 = disagree, 3 = neither, 4 = agree, 5 = strongly agree) that assess an individual’s feelings toward their body weight (e.g., During the past few weeks, my [name of bowel condition] has made it difficult for me to keep my weight where I’d like it to be.]). Items were averaged, with higher mean scores indicating negative body image.

The Relationships/Social Interference scale has 7 items on a 5-point scale (1 = strongly disagree, 2 = disagree, 3 = neither, 4 = agree, 5 = strongly agree) that assess interpersonal relationships and enjoyment of life through socializing (e.g., “During the past few weeks, I have been embarrassed by my need to be near a bathroom or the smell caused by my bowel problems.”). Items were averaged, with higher mean scores indicating greater social interferences and poor relationships with others.

The Health Worry/Future Concerns scales has 5 items on a 5-point scale (1 = strongly disagree, 2 = disagree, 3 = neither, 4 = agree, 5 = strongly agree) that assess concerns with future careers and longevity (e.g., “I worry about losing control of my bowels.”). Items were averaged, with higher mean scores indicating more health worries and future concerns.

Another reliable and validated measure that was used to assess health-related quality of life is a shortened version of the Centers for Disease Control and Prevention Heath-Related Quality of Life measure (CDC HRQOL-14) [37]. Only items 2 and 3 on this instrument were used to calculate a summary index of unhealthy days in the past month, with a logical maximum of 30 unhealthy days.

#### 2.2.3. Eating Disorder Examination Questionnaire (EDE-Q)

The Eating Disorder Examination Questionnaire (EDE-Q) is a version of the Eating Disorder Examination (EDE) that is self-reported [38]. The EDE-Q was designed to target limitations of the EDE, which is conducted in a semi-structured interview requiring a trained assessor [39,40]. The self-report EDE-Q is a reliable and valid measure used to assess eating disorder risk in a series of 28 questions and consists of four subscales [41]. The restraint eating subscale assesses an individual’s attempts to restrict food intake to affect their weight and shape. The eating concern subscale assesses the level of concern about eating. The weight and shape concern subscales measure, respectively, an individual’s level of concern about their weight and shape, and how strongly these concerns impact their self-evaluation. Each question is rated along a 7-point scale that ranges from 0 to 6 (0 = no days, 1 = 1–5 days, 2 = 6–12 days, 3 = 13–15 days, 4 = 16–22 days, 5 = 23–27 days, 6 = everyday) to assess the frequency of eating disorder symptoms. Other items are scored on a 7-point scale from “not at all” to “markedly” to assess the severity of symptoms, or they are fill-in-the-blank responses to assess the number of times the symptoms have occurred within the past 28 days. An overall global EDE-Q score is then calculated from the average of the four subscales. Scores ≥ 4 indicate increased eating disorder risk.

A single item from the EDE-Q [38], “Over the past four weeks (28 days), how many days have such incidents occurred (i.e., you have eaten an unusually large amount of food given the circumstances and had a sense of loss of control at the time)” was used to determine the frequency of binge episodes in the past month. Responses were dichotomized into those who reported a binge episode ≥ 4 times in the past 28 days.

Inappropriate compensatory behavior items from the EDE-Q [38] included the frequency over the past 28 days of self-induced vomiting, laxative/medicine misuse, and/or excessive exercise to control one’s body weight and shape. Responses for self-induced vomiting and laxative/medicine misuse were categorized into four or more times of participating in each of these behaviors over the past 28 days, while excessive exercising was categorized into 16 or more times of participating in this behavior over the past 28 days. These cut-off scores indicate greater eating disorder risk.

#### 2.2.4. Three-Factor Eating Questionnaire (TFEQ-18)

The Three-Factor Eating Questionnaire (TFEQ-18) is a reliable and valid measure that was used to assess only the disinhibited eating (3 items), and emotional eating (3 items) scales [42,43]. The disinhibited eating scale assesses the tendency to overeat and having limited self-regulation to control food intake (e.g., “Sometimes when I start eating, I just can’t seem to stop.”), while the emotional eating scale assesses the tendency to eat in response to negative mood (e.g., “When I feel lonely, I console myself by eating.”). All items are on a 4-point scale (definitely false = 1, mostly false = 2, mostly true = 3, definitely true = 4). Items on each scale were averaged for a mean score, with higher scores indicating greater emotional eating and disinhibited (uncontrolled) eating.

### 2.3. Data Analysis

The internal consistency of these scales was assessed, as applicable, using Cronbach alpha coefficients. Descriptive statistics of sociodemographic and health characteristics as well as QOL and maladaptive eating behaviors were stratified by gender in young adults with bowel conditions of IBS and IBD. Mann–Whitney U (non-parametric) tests for continuous variables and Chi-square tests for categorical variables examined significant QOL and maladaptive eating behavior differences between men and women with bowel conditions. Additionally, partial correlation coefficients controlling for age of bowel condition diagnosis were performed to explore linear associations among maladaptive eating behaviors and QOL. Statistical significance was set at *p* < 0.05. All analyses were performed using SPSS version 28 (Chicago, IL, USA).

## 3. Results

Participants (*N* = 73) were, on average, in their mid-20s and mostly women (69.9%) (Table 1). Additionally, most participants reported being enrolled as graduate students (39.73%). Interestingly, almost one-third (30.14%) identified as being Hispanic, with most participants identifying as white (73.97%). Men had a mean body mass index (27.17 ± 7.04 SD) above the healthy body mass index range (BMI 18.5 to <25) indicating overweight status (BMI 25 to <30). Women had a mean body mass index (24.36 ± 4.68 SD) within the healthy range, but close to the upper limit.

Among men (*n* = 22), half reported being diagnosed with Crohn’s disease, almost one-third with ulcerative colitis, and over one-quarter with irritable bowel syndrome (see Table 1). For women (*n* = 51), less than a quarter reported being diagnosed with Crohn’s disease, over a third with ulcerative colitis diagnosis, and more than half with irritable bowel syndrome. The self-reported mean age for diagnosis of a bowel condition occurred during late adolescence, with men reporting a slightly younger age of diagnosis compared to women. None of the men reported having a history of an eating disorder while 13.73% of women reported a history of an eating disorder.

The internal reliability of the QOL scales, as measured by Cronbach alpha coefficients, ranged from fair to excellent, except for the body image (α = 0.65) and health worry/future concern (α = 0.58) scales, which were poor (Table 2). On a 5-point scale from 1 to 5 with higher mean scores indicating poorer quality of life, the mean global quality of life score (3.13 ± 0.65 SD) for all participants, as well as the six quality of life subscales, revealed participants had some discontent with their quality of life. The average total number of physically and mentally unhealthy days reported in the past month was elevated (10.72 ± 7.46 SD days). Mann–Whitney U tests revealed no significant quality of life mean score differences between men and women.

The internal reliability of the Eating Disorder Examination Questionnaire (EDE-Q) scales, as measured by Cronbach alpha coefficients, ranged from good to excellent (Table 3). Global and subscale mean scores on the EDE-Q were on the lower end of the 0-to-6 possible score range, where a score of 4 or higher indicates increased eating disorder risk. However, the global and subscale EDE-Q mean scores were higher than normative EDE-Q data found in healthy young adult men and women [44]. Mann–Whitney U tests revealed no significant EDE-Q global or subscale mean differences between men and women.

Frequent binge episodes, classified as four or more binge eating episodes in the past 28 days, revealed that more than half (58.90%) had experienced binge eating episodes in the past 28 days (see Table 3). Almost half reported self-induced vomiting (41.10%) and medicine misuse (45.21%) four or more times in the past month, with a smaller percentage that endorsed excessive exercising (10.96%) 16 or more times in the past month as a compensatory behavior. Fisher Exact tests revealed no proportional gender differences in the excessive exercising compensatory behavior. However, men reported using self-induced vomiting (68.18% vs. 29.41%) and medicine misuse (63.64% vs. 37.25%) at a significantly higher proportion than women.

Cronbach alpha coefficients for disinhibited eating (α = 0.68) and emotional eating scales (α = 0.78) were fair (see Table 3). Mean scores indicated some emotional and disinhibited eating in all participants. However, Mann–Whitney U tests revealed no significant gender differences in mean disinhibited and emotional eating scales.

Partial correlations controlling for age of bowel condition diagnosis revealed significant linear associations between QOL and some maladaptive eating behaviors that were moderate in strength (Table 4). That is, the Global EDE-Q score, an indicator for eating disorder risk, was positively associated with the QOL Global score (*r_partial_* = 0.534, *p* < 0.001) and total number of unhealthy days reported in the past month (*r_partial_* = 0.452, *p* < 0.001).

## 4. Discussion

Findings from this exploratory cross-sectional study suggest that both men and women with bowel conditions (IBS and IBD) may experience poor QOL and exhibit maladaptive eating behaviors, which may increase their risk of developing an eating disorder. Additionally, a high proportion of women with bowel conditions reported a history of an eating disorder (13.7%). As a preventative approach, those with bowel conditions may benefit from eating disorder risk screenings as part of their routine medical care, given the moderately strong correlations found between maladaptive eating behaviors and poor quality of life.

More than 1 out of 10 women with a bowel condition reported having a history of an eating disorder, whereas none of the men reported having a history of an eating disorder, which is consistent with the general population [45]. The median age of onset for binge eating disorder is about 21 years of age, and for both bulimia nervosa and anorexia nervosa, the median age of onset is about 18 years old [45]. Compared to the age of diagnosis of a bowel condition reported by participants in our study (~16 years), this is before the median age of diagnosis of most eating disorders, but casual inferences cannot be made given the cross-sectional nature of this study. Disordered eating behaviors can also develop for years before the clinical diagnosis of a bowel condition. Thus, it is unclear whether a bowel condition develops prior to having an eating disorder or vice versa, or if they develop simultaneously.

Compared to normative EDE-Q data from a healthy U.S. college population, those with bowel conditions in our study had higher mean EDE-Q global scores [44]. For example, the mean EDE-Q global score for men (2.49 ± 1.26 SD vs. 0.95 ± 0.98 SD) and women (2.30 ± 1.12 SD vs. 1.65 ± 1.30 SD) with bowel conditions was higher compared to the healthy population of young adults [44], respectively, suggesting those with bowel conditions may exhibit more maladaptive eating behaviors. Consistent with prior work, young adults with bowel conditions may be at greater risk for disordered eating because of the practices used in managing their diseases. However, in a cross-sectional study among IBD participants (*N* = 50; 68% women; mean age ~17 years), the mean global EDE-Q score was 1.79 ± 1.56 SD which is lower than the mean score (2.36 ± 1.16 SD) found in our study [46]. Regardless of the inconsistencies in the normative EDE-Q findings among prior studies of bowel condition populations, our study is still consistent in that those with bowel conditions tend to score higher on the EDE-Q compared to healthy populations, potentially placing them at increased risk for an eating disorder.

Prior work has found that those with DRCHCs like IBS and IBD are more likely to misuse medicine and exercise excessively than healthy participants [31]. In our study, medicine misuse approached a statistically significant difference between men and women, with proportionally more men reporting this behavior than women. However, normative data among healthy young adults reported women use self-induced vomiting and medicine misuse as compensatory behaviors more frequently than men [44]. Findings from a more recent cross-sectional study (*N* = 9713) found similar uses of compensatory behaviors among healthy undergraduate and graduate students (men 29%; women 31%), apart from binge eating behaviors, which were higher among women (49%) than men (30%) [47]. This pattern of high binge eating among women is consistent with other healthy young adult populations [44]. However, 58.90% of bowel condition participants in our study reported binge eating episodes occurring four or more times in the past 28 days, which is higher than prior studies among healthy young adult populations. Perhaps the pressure placed on them by their disease management to maintain a healthy body weight, as well as changes to their outward appearance due to medical complications (e.g., unintentional weight loss or gain) make those with bowel conditions more susceptible to endorsing compensatory behaviors to control their body weight and shape [16,48].

For those with bowel conditions, exercise may be recommended by healthcare professionals, as it has been shown to improve many symptoms associated with these diseases [49]. However, in our study, more than one out of ten men with a bowel condition reported engaging in excessive exercise more than 16 times in the past month. Proportionally there were fewer women that reported excessive exercise, although this difference was not statistically significant. This finding is congruent with healthy populations, where men are more likely to use excessive exercise than women to control their weight and shape [44,50]. In western culture, the sociocultural ideals of appearance and associated pressures that are internalized, leading to body dissatisfaction and disordered eating, may partly explain the gender differences found in our study [51]. That is, women are primarily faced with sociocultural pressure to be thin, while men face sociocultural pressure to be lean and muscular [52]. The pursuit of these ideals can lead to maladaptive eating behaviors that can be of concern in those with bowel conditions.

Prior work assessing QOL in those with bowel conditions has presented results for IBS and IBD separately. In a systematic review that included 17 research articles, it was reported that in a population with IBS that received consultation for their condition, HRQOL was poorer compared to a healthy adult population [53]. Among a sample of 314 participants with IBD, HRQOL was poorer when compared to the general population [54]. A common theme among QOL measures in IBD and IBS populations is that severity of disease symptoms is indicative of lower HRQOL scores, but this does not account for differences in gender. In a cross-sectional study of IBD participants (*N* = 134; 81.3% female; mean age 32.5 years; 94% Caucasian), women were found to have poorer HRQOL than men, as reported on the inflammatory bowel disease questionnaire [55]. However, in a review article examining 107 research articles, QOL gender differences in those with IBD were mixed, with most finding lower QOL in women, but some finding lower QOL in men [56]. In our study of both IBS and IBD participants, there were no gender differences in QOL measures. In general, men and women with bowel conditions may both be at risk for poor quality of life due to the interference that their disease may impose on normal physical and psychological functions. Additionally, although severity of disease symptoms was not assessed in our study, it may be speculated that participants in our study had greater disease symptoms present, given the average number of unhealthy days reported was, on average, 11 days in the past month.

When comparing HRQOL between patients with eating disorders and the healthy population, the differences are considerable. In a review article that examined HRQOL among those with eating disorders, there was a reduced HRQOL [57]. Even among patients not meeting all DSM diagnostic criteria for an eating disorder, purging behaviors, as well as motivations for exercising predicted HRQOL scores [57]. Similarly, in our study there were positive linear associations that were moderate in strength between maladaptive eating behaviors and QOL, even after controlling for age of bowel condition diagnosis. This is alarming, given the negative impact maladaptive eating behaviors may have on treatment of bowel conditions and overall QOL.

The strengths and limitations of our study are worth noting. A limitation of our cross-sectional study is that only correlation, not causation, can be established. In addition, our study had a small sample, with a majority (70%) being women. As a result of a small sample size, findings from this study cannot be generalized to a broader population of young adults with these bowel conditions. While most survey questions used in this study are reliable and valid measures, some of the measures like the EDE-Q may not be suitable for those with IBS and IBD. For instance, the EDE-Q may falsely give higher scores due to the nature of how the questions are phrased for the healthy population. Additionally, as the disease-specific QOL measure lacks validation in populations with bowel diseases, the interpretation of these findings should be approached cautiously. Finally, when conducting survey-based research, there is always a chance of response and recall bias, especially when participants are asked to remember behaviors over the past month.

Importantly, a strength of this study is that it adds to the limited research exploring quality of life, maladaptive eating behaviors, and gender differences in those with IBS and IBD. Despite the small sample size, the population in our study was relatively diverse. Another strength of this study was that it allowed participants to remain anonymous, which may have resulted in more accurate responses on sensitive topics surrounding bowel conditions and disordered eating behaviors. Future research with a larger sample size should be conducted to validate the results of this study among young people with bowel conditions. Additionally, given the unique characteristics of the different bowel conditions, it would be helpful to analyze gender differences, maladaptive eating behaviors, and quality of life measures by bowel condition type (IBS and IBD) in future research.

### Implications for Clinical Practice

There are clinical implications from these research findings worth noting. Gastroenterologists, registered dietitian nutritionists, primary care physicians, pediatricians, and mental health providers all play a role in identifying and addressing maladaptive eating behaviors in patients with bowel conditions. Routine screening and early referral to nutrition and behavioral health specialists can help prevent the progression to a clinical eating disorder. For parents and caregivers, particularly of adolescents or young adults with bowel diseases, awareness of maladaptive eating behaviors and eating disorders is equally important. Caregivers may be the first to notice concerning behaviors such as excessive dietary restriction, food-related anxiety, or rigid eating rules that may appear to be symptom management for their bowel condition but may in fact be a signal of maladaptive eating. Open communication from family and caregivers and timely involvement of healthcare professionals can help families support healthy eating patterns and reduce the risk of progression to an eating disorder.

Ultimately, these findings emphasize the importance of a collaborative, preventative approach to monitoring and researching the intersection of bowel diseases and maladaptive eating behaviors. Expanding research on dietary interventions, combined with interdisciplinary care and caregiver involvement, is critical to protecting both the nutritional status and overall well-being of individuals with bowel conditions.

## 5. Conclusions

Overall, our findings suggest that those with bowel conditions may be at risk for maladaptive eating behaviors and poor quality of life. There are physical and mental health implications for individuals with bowel conditions that may affect their body satisfaction, quality of life, and eating behaviors. More research is warranted in examining the potential relationship between the development of eating disorders and bowel conditions such as IBS and IBD. While further research is needed, our study brings to light the implications of living with a bowel condition and the need to potentially screen for eating disorder risk as part of routine medical treatment in this population.

## Figures and Tables

**Table 1:** Sociodemographic & health characteristics of bowel condition participants (*N* = 73).

Characteristic	All Participants (*N* = 73)	Men (*n* = 22)	Women (*n* = 51)
	Mean ± SD or *N* (%)	Mean ± SD or *N* (%)	Mean ± SD or *N* (%)
Age (years)	25.16 ± 2.94	25.09 ± 3.56	25.20 ± 2.67
Age of bowel condition diagnosis (years)	16.33 ± 7.65	13.64 ± 7.61	17.49 ± 7.45
Body Mass Index	25.10 ± 5.48	27.17 ± 7.04	24.36 ± 4.68
Racial Identity			
Asian	2 (2.74)	0 (0.00)	2 (3.92)
Black or African American	4 (5.48)	1 (4.55)	3 (5.88)
White	54 (73.97)	16 (72.73)	38 (74.51)
Other	13 (17.81)	5 (22.73)	8 (15.69)
Hispanic (% yes)	22 (30.14)	9 (40.91)	13 (25.49)
Year in College			
Freshman	2 (2.74)	2 (9.09)	0 (0.00)
Sophomore	5 (6.85)	2 (9.09)	3 (5.88)
Junior	4 (5.48)	3 (13.64)	1 (1.96)
Senior	16 (21.92)	2 (9.09)	14 (27.45)
Graduate Student	29 (39.73)	9 (40.91)	20 (39.22)
Not enrolled student	13 (17.81)	2 (9.09)	11 (21.57)
Other	4 (5.48)	2 (9.09)	2 (3.92)
Bowel Condition Diagnosis			
Crohn’s Disease (% yes)	20 (27.40)	11 (50.00)	9 (17.65)
Ulcerative Colitis (% yes)	26 (35.62)	7 (31.82)	19 (37.25)
Irritable Bowel Syndrome (% yes)	35 (47.95)	6 (27.27)	29 (56.86)
History of Eating Disorder (% yes)	7 (9.59)	0 (0.00)	7 (13.73)

**Table 2:** Quality of life characteristics of bowel condition participants (*N* = 73).

		All Participants (*N* = 73)	Men (*n* = 22)	Women (*n* = 51)			
Characteristic	Cron-Bach’s α	Mean ± SD or *N* (%)	Mean ± SD or *N* (%)	Mean ± SD or *N* (%)	Mann–Whitney U *	95% CI ^†^	*p*
Quality of Life (Global Score) ^a^	0.94	3.13 ± 0.65	3.03 ± 0.59	3.17 ± 0.68	611.0	−0.40–0.17	0.547
Dysphoria/Emotional ^b^	0.89	3.03 ± 0.77	2.94 ± 0.63	3.08 ± 0.83	590.0	−0.50–0.30	0.727
Interference with Activity of Physical Functioning ^c^	0.90	3.13 ± 0.84	3.11 ± 0.73	3.13 ± 0.89	545.5	−0.38–0.38	0.852
Food Avoidance ^d^	0.83	3.32 ± 0.97	3.15 ± 0.92	3.39 ± 0.99	621.5	−0.67–0.33	0.463
Body Image ^e^	0.65	3.05 ± 0.98	2.98 ± 0.99	3.08 ± 0.99	587.0	−0.50–0.50	0.752
Relationship/Social Interference ^f^	0.82	3.21 ± 0.79	2.99 ± 0.73	3.30 ± 0.81	652.0	−0.71–0.14	0.273
Health Worry/Future Concern ^g^	0.58	3.14 ± 0.65	3.07 ± 0.48	3.16 ± 0.71	597.0	−0.40–0.20	0.663
Number of Unhealthy Days in Past Month ^h^	n/a	10.72 ± 7.46	10.95 ± 6.18	10.62 ± 8.00	509.0	−2.50–5.00	0.532

* Mann–Whitney U (non-parametric) tests examined significant quality of life characteristic differences between men and women. ^†^ 95% confidence interval based on Hodges–Lehman median differences. ^a^ Average score of 35 items on 5-point scale, with higher mean scores indicating poor quality of life. ^b^ Average score of 10 items on 5-point scale (1 = never, 2 = seldom, 3 = sometimes, 4 = often, and 5 = all the time), with higher mean scores indicating greater emotional vulnerability. ^c^ Average score of 8 items on a 5-point scale (1 = never, 2 = seldom, 3 = sometimes, 4 = often, and 5 = all the time), with higher mean scores indicating greater interference with activities. ^d^ Average score of 3 items on a 5-point scale (1 = strongly disagree, 2 = disagree, 3 = neither, 4 = agree, 5 = strongly agree), with higher mean scores indicating greater food avoidance. ^e^ Average scores of 2 items on a 5-point scale (1 = strongly disagree, 2 = disagree, 3 = neither, 4 = agree, 5 = strongly agree), with higher mean scores indicating negative body image. ^f^ Average scores of 7 items on a 5-point scale (1 = strongly disagree, 2 = disagree, 3 = neither, 4 = agree, 5 = strongly agree), with higher mean scores indicating greater social interferences and poorer relationships with others. ^g^ Average score of 5 items on a 5-point scale (1 = strongly disagree, 2 = disagree, 3 = neither, 4 = agree, 5 = strongly agree), with higher mean scores indicating more health worries and future concerns. ^h^ Average score of 2 items indicating a summary index of unhealthy physical and mental days in the past month, with a logical maximum of 30 unhealthy days.

**Table 3:** Maladaptive eating behaviors of bowel condition participants (*N* = 73).

		All Participants (*N* = 73)	Men (*n* = 22)	Women (*n* = 51)	Mann–Whitney U		
Characteristic	Cronb-ach’s α	Mean ± SD or *N* (%)	Mean ± SD or *N* (%)	Mean ± SD or *N* (%)	or Fisher Exact Tests *	95% CI ^†^	*p*
Eating Disorder Examination Questionnaire ^a^							
Restraint Eating	0.89	1.83 ± 1.32	2.06 ± 1.45	1.73 ± 1.26	482.0	−0.40–1.20	0.341
Eating Concerns	0.80	2.00 ± 1.20	2.25 ± 1.42	1.89 ± 1.09	455.0	−0.20–1.20	0.202
Shape Concerns	0.90	2.68 ± 1.37	2.69 ± 1.41	2.68 ± 1.37	536.0	−0.50–0.75	0.764
Weight Concerns	0.78	2.61 ± 1.34	2.74 ± 1.35	2.56 ± 1.35	496.5	−0.40–1.00	0.437
Global EDE-Q ^b^		2.36 ± 1.16	2.49 ± 1.26	2.30 ± 1.12	488.0	−0.32–0.91	0.380
Binge Episodes ^c^							
≥4x in past 28 days	n/a	43 (58.90)	16 (72.73)	27 (52.94)	2.49	n/a	0.130
Inappropriate Compensatory Behaviors in Past Month (% yes) ^d^							
≥4x Self-Induced Vomiting	n/a	30 (41.10)	15 (68.18)	15 (29.41)	9.54	n/a	0.004
≥4x Medicine Misuse	n/a	33 (45.21)	14 (63.64)	19 (37.25)	4.31	n/a	0.045
≥16x Excessive Exercising	n/a	8 (10.96)	3 (13.64)	5 (9.80)	0.23	n/a	0.454
Three Factor Eating Questionnaire							
Disinhibited Eating ^e^	0.68	2.44 ± 0.66	2.39 ± 0.59	2.46 ± 0.69	604.0	−0.33–0.33	0.599
Emotional Eating ^f^	0.78	2.59 ± 0.69	2.58 ± 0.65	2.60 ± 0.71	564.0	−0.33–0.33	0.971

* Mann–Whitney U or Fisher Exact Tests examined significant maladaptive eating behavior differences between men and women. ^†^ 95% confidence interval based on Hodges–Lehman median differences. ^a^ Average scores of 28 items on a 7-point scale from 0 to 6 (0 = no days, 1 = 1–5 days, 2 = 6–12 days, 3 = 13–15 days, 4 = 16–22 days, 5 = 23–27 days, 6 = everyday), with higher scores indicating greater frequency of eating disorder symptoms. ^b^ Average of four EDE-Q subscales, with mean scores ≥ 4 indicating increased eating disorder risk. ^c^ Single item from EDE-Q used to determine frequency of binge eating episodes dichotomized into those with ≥4 binge eating episodes in the past 28 days. ^d^ Dichotomized based on 4 or more times self-induced vomiting or medicine misuse was endorsed over the past 28 days and 16 or more times excessive exercising was endorsed as a means to control one’s body shape and/or weight. ^e^ Average score of 3 items on a 4-point scale (definitely false = 1, mostly false = 2, mostly true = 3, definitely true = 4), with higher scores indicating greater disinhibited (uncontrolled) eating. ^f^ Average score of 3 items on a 4-point scale (definitely false = 1, mostly false = 2, mostly true = 3, definitely true = 4), with higher scores indicating greater emotional eating.

**Table 4:** Partial correlation coefficients of maladaptive eating behaviors and QOL (*N* = 73).

Characteristic ^a^	1	2	3	4	5
QOL Global Score	---				
Number of Unhealthy Days in past month	0.527 ***	---			
Disinhibited Eating	0.274 *	0.108	---		
Emotional Eating	0.175	0.131	0.701 ***	---	
Global EDE-Q Score	0.534 ***	0.452 ***	0.407 ***	0.357 **	---

^a^ Partial correlations controlling for age of bowel condition diagnosis. * *p* < 0.05, ** *p* < 0.01, *** *p* < 0.001.

## Data Availability

The data presented in this study are available on request from the corresponding author.
